# Anxiety, depression, and negative affect in women with and without fertility issues: a network comparison study

**DOI:** 10.1192/j.eurpsy.2025.425

**Published:** 2025-08-26

**Authors:** N. Ćirović, J. Opsenica Kostić, M. Mitrović, M. Guberinić, M. Spasić Šnele, I. Janković, M. Trenkić

**Affiliations:** 1 Department of Psychology, Faculty of Philosophy, University of Niš; 2 Department of Gynecology and Obstetrics, Faculty of Medicine, University of Niš, Niš, Serbia

## Abstract

**Introduction:**

Infertility is a biopsychosocial crisis. While there are studies demonstrating heightened negative affect (e.g., depression and anxiety) in women undergoing in vitro Fertilization (IVF), the findings are still inconsistent. The network paradigm allows for a more in-depth examination of symptom dynamics behind specific psychopathological states. A recent development allows one to compare networks from different groups using three characteristics: global strength (overall level of network node connectivity), edge strength (level of association between symptoms), and network structure (comparing all edges in the network across two groups).

**Objectives:**

This study aims to compare the networks of anxiety, depression, and negative affect across women who have fertility issues or undergoing IVF and women without these issues.

**Methods:**

Sample 1 consisted of 197 women with fertility issues (age: M = 37.73, SD =5.13) and 370 women without such issues (age: M = 36.25, SD = 6). Sample 2 consisted of 205 women undergoing IVF (M = 40; SD = 5.29) and 222 mothers without fertility issues (M=28; SD = 4. 93). Sample 3 consisted of 162 women undergoing IVF (M= 35.58; SD=5.04) and 129 mothers without fertility issues (M= 34.37; SD= 4.94). PHQ-9 (Patient Health Questionnaire; depression measure) was administered to the sample 1, GAD (generalized anxiety disorder measure) was administered to sample 2, and PANAS - NA (negative affect measure) was administered to sample 3. NetworkComparisonTest R package was used to compare the networks. EBICglasso was used to estimate the networks.

**Results:**

Regarding the depression symptoms (sample 1; image 1) - the networks across the two groups are highly similar with respect to overall connectivity (S =.051; p = .73) and overall network structure (M = .16, p =.87). Regarding generalized anxiety symptoms (sample 2; image 2), the findings are replicated with overall connectivity being the same across the two groups (S =.10, p = .34) and network structure being the same across the two groups (M = .28, p= .09). Finally, the negative affect (sample 3; image 3) network connectivity (S = .02, p = .93) and network structure (M = .23, p = .53) are identical across the two groups.

**Image 1:**

**Figure:**
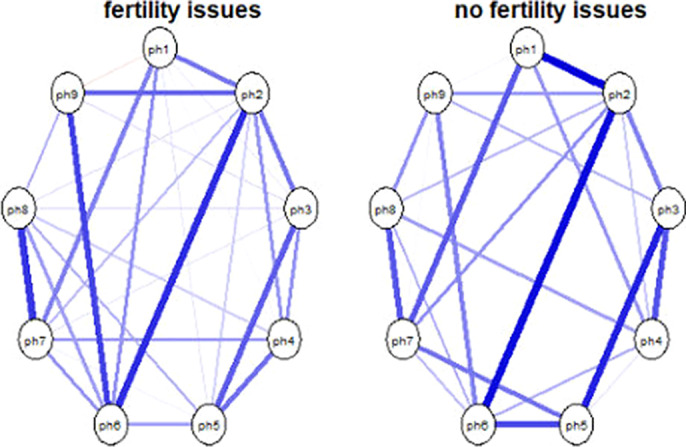

**Image 2:**

**Figure:**
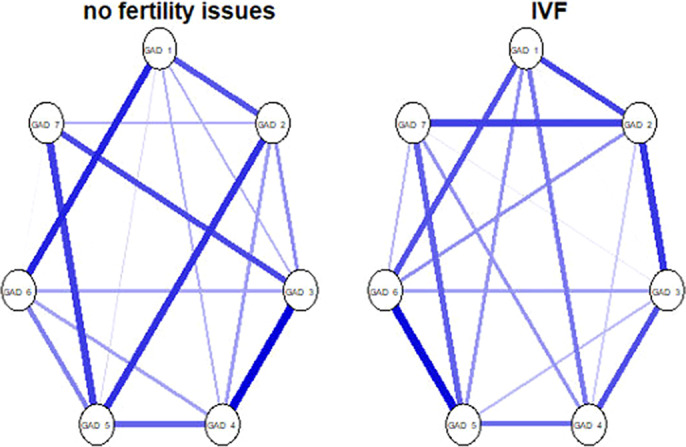

**Image 3:**

**Figure:**
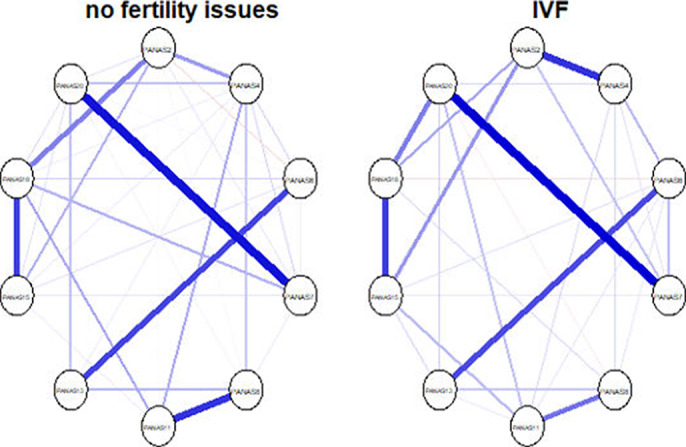

**Conclusions:**

The networks of negative affect, depression, and anxiety are highly similar across women with fertility issues and women without such issues. Therefore, fertility issues do not seem to affect the structure of symptoms of depression, anxiety, and negative affect. Finally, it is argued here that the knowledge of these disorders (and negative affect) can be generalized to the population of women who have fertility issues.

**Disclosure of Interest:**

N. Ćirović Grant / Research support from: Nikola Ćirović was supported by the Science Fund of the Republic of Serbia, #GRANT No 1568, Identity Crisis in Women Facing Infertility: Mixed Methods Approach – InsideMe., J. Opsenica Kostić Grant / Research support from: Jelena Opsenica Kostić was supported by the Science Fund of the Republic of Serbia, #GRANT No 1568, Identity Crisis in Women Facing Infertility: Mixed Methods Approach – InsideMe., M. Mitrović Grant / Research support from: Milica Mitrović was supported by the Science Fund of the Republic of Serbia, #GRANT No 1568, Identity Crisis in Women Facing Infertility: Mixed Methods Approach – InsideMe., M. Guberinić Grant / Research support from: Mila Guberinić was supported by the Science Fund of the Republic of Serbia, #GRANT No 1568, Identity Crisis in Women Facing Infertility: Mixed Methods Approach – InsideMe., M. Spasić Šnele Grant / Research support from: Miljana Spasić Šnele was supported by the Science Fund of the Republic of Serbia, #GRANT No 1568, Identity Crisis in Women Facing Infertility: Mixed Methods Approach – InsideMe., I. Janković Grant / Research support from: Ivana Janković was supported by the Science Fund of the Republic of Serbia, #GRANT No 1568, Identity Crisis in Women Facing Infertility: Mixed Methods Approach – InsideMe., M. Trenkić Grant / Research support from: Milan Trenkić was supported by the Science Fund of the Republic of Serbia, #GRANT No 1568, Identity Crisis in Women Facing Infertility: Mixed Methods Approach – InsideMe.

